# Atherosclerosis Prevalence among Different Physical Activity Patterns in Adult Men

**DOI:** 10.3390/jcm13175062

**Published:** 2024-08-26

**Authors:** Jose Luis Perez-Lasierra, Jose Antonio Casajús, Alejandro Gonzalez-Agüero, Jose Antonio Casasnovas, Carolina Torrijo-Blanche, Sofia Gimeno-Ruiz, Belén Moreno-Franco

**Affiliations:** 1 Facultad de Ciencias de la Salud, Universidad San Jorge, 50830 Villanueva de Gállego, Spain; jlperez@usj.es; 2 EXER-GENUD (Growth, Exercise, Nutrition and Development) Research Group, 50009 Zaragoza, Spain; joseant@unizar.es (J.A.C.); alexgonz@unizar.es (A.G.-A.); 3 Department of Physiatry and Nursing, Universidad de Zaragoza, 50009 Zaragoza, Spain; 4 Centro de Investigación Biomédica en Red de Fisiopatología de la Obesidad y Nutrición (CIBEROBN), 28029 Madrid, Spain; 5 Department of Medicine, Psiquiatry and Dermatology, Universidad de Zaragoza, 50009 Zaragoza, Spain; jacasas@unizar.es; 6 Instituto de Investigación Sanitaria de Aragón (IIS Aragón), 50009 Zaragoza, Spain; 7 Centro de Investigación Biomédica en Red de Enfermedades Cardiovasculares (CIBERCV), 28029 Madrid, Spain; 8 Department of Microbiology, Pediatrics, Radiology and Public Health, Universidad de Zaragoza, 50009 Zaragoza, Spain; carolinatorrijob@gmail.com; 9 Faculty of Veterinary, Universidad de Zaragoza, 50009 Zaragoza, Spain; gimenoruizsofia@gmail.com

**Keywords:** subclinical atherosclerosis, exercise, physical activity intensity

## Abstract

**Background:** Physical activity (PA) intensity could play a key role in atherosclerosis risk, but the existing literature shows controversial results. The aim of this study was to analyze the association of different PA levels with the presence of subclinical atherosclerosis in femoral and carotid arteries. **Methods:** A cross-sectional analysis was conducted of 449 middle-aged men belonging to the Aragon Workers’ Health Study. Demographic, anthropometric, and clinical data were obtained during the annual medical examination. Ultrasonography was used to assess the presence of atheroma plaques in femoral and carotid territories. Accelerometry was used to assess habitual PA. Participants were categorized into vigorous PA (VPA) groups (0 min/week, >0–60 min/week, >60 min/week), and into moderate to vigorous PA (MVPA) groups using terciles as cut-offs. **Results:** Compared with participants who completed 0 min/week of VPA, those participants who completed >60 min/week of VPA had fully adjusted odds of subclinical atherosclerosis of 0.47 (95%CI: 0.22, 0.99, *p* < 0.05) and 0.35 (95%CI: 0.17, 0.73, *p* < 0.05) for femoral and any territory (femoral and/or carotid) respectively. No significant differences were observed in the prevalence of atheroma plaques in any vascular territory between the different MVPA groups. **Conclusions:** Performing more than 60 min/week of VPA is associated with reduced odds for subclinical atherosclerosis in femoral or any vascular territory in adult men.

## 1. Introduction

Atherosclerosis is a chronic inflammatory disease of blood vessels, which is highly present among middle-aged individuals, reaching a prevalence of higher than 70% in males [1,2]. Atherosclerosis constitutes a multifactorial process modulated by several non-modifiable factors, such as genetics, but also by modifiable elements like smoking, deleterious dietary patterns, or physical inactivity [2,3,4].

Physical activity (PA) is a key factor in the prevention and control of cardiovascular disease (CVD). The health benefits associated with PA are clear and well-documented in the literature [5,6]. The most recent worldwide PA guidelines support the recommendation that adults should undertake at least 150 min of moderate-intensity or 75 min of vigorous-intensity aerobic PA per week to improve their general health [6], supporting that any PA is better than none, even light-intensity PA [6]. Several studies have demonstrated its anti-inflammatory and anti-atherogenic effects [3,7,8,9,10,11]; however, as evidenced by other studies, this role remains contradictory [12,13,14,15]. Lifelong high doses of exercise (>2000 MET-min/week) or performing very vigorous-intensity exercise (≥9 METs) have been associated with a significantly higher risk of carotid atherosclerosis [12]. On the other hand, a recent study found that PA was inversely associated with the presence of atheroma plaques and also that a higher level of PA (≥8320 MET-min/week) was associated with a lower risk of subclinical atherosclerosis [11].

The PA level, understood as a combination of duration and intensity, may play a key role in understanding the relationship between PA and atherosclerosis. PA intensity modulates the secretion of catecholamines, hormones that control the cardiac response to activity. At high PA intensities, catecholamine levels increase exponentially [16], causing heart rate and blood pressure to rise, which may promote an atherogenic effect through turbulent blood flow. Indeed, the literature has demonstrated that moderate-intensity exercise is associated with an anti-inflammatory effect [17], while at higher intensities, the increase in plasma catecholamine concentrations can have a pro-inflammatory effect [18].

Considering the existing controversy in the current scientific literature, the aim of this study was to analyze the association of different PA levels with the presence of femoral and carotid atherosclerosis in a sample of middle-aged male factory workers from Spain, free of clinical CVD.

## 2. Materials and Methods

### 2.1. Study Design and Participants

This cross-sectional analysis was carried out in a subsample of participants belonging to the Aragon Worker’s Health Study (AWHS) [19]. The AWHS is a prospective cohort aiming to investigate the risk and protective factors of the development and progression of subclinical atherosclerosis in 5678 workers of a car manufacturing factory. From 2011 to 2014, 449 workers, aged between 39 and 59 years and apparently free of CVD, were invited to undergo subclinical atherosclerosis imaging and PA monitorization and to complete questionnaires on diet, behavior, and lifestyle factors. We excluded women (n = 10) and those with missing data on relevant variables (n = 11), so the final sample was composed of 428 men. The study protocol conforms to the ethical guidelines of the 1975 Declaration of Helsinki and was approved by the Clinical Research Ethics Committee of Aragon (CEICA PI07/09). Written informed consent was obtained from each participant included in the study.

### 2.2. Physical Activity Assessment

The assessment of PA was performed using an ActiGraph GT3X+ accelerometer (ActiGraph, Pensacola, FL, USA). Participants were required to wear the device on the right hip for 7 consecutive days. Accelerometers were initialized to record accelerations at 30 Hz with a dynamic range of ±6 G. The raw records were downloaded and processed with AcliLife v.6.13.4 software (ActiGraph, Pensacola, FL, USA). Time in different PA intensities was classified using the thresholds for adults previously proposed by Freedson using the vector magnitude [20]: (a) light PA (LPA): 0–2690 counts/min, (b) moderate PA (MPA): 2691–6166 counts/min, (c) vigorous PA (VPA): 6167–9642 counts/min, (d) very vigorous PA (VVPA): ≥9643 counts/min, (e) moderate to vigorous PA (MVPA): ≥2691 counts/min. Participants’ records were considered valid when they covered at least 10 h/day during ≥4 days, requiring at least 3 weekdays and 1 weekend day.

Finally, the participants were classified into different groups based on their VPA level (0 min/week; >0–60 min/week; >60 min/week), and also in different groups based on terciles of MVPA (T3: ≤340 min/week; T2: 340.01–497 min/week; T1: ≥497.01 min/week). We propose these PA groups because the previously defined PA groups, based on PA recommendation guidelines [6], produced skewed group distribution. For the 75 min/week of VPA cut-off, the distribution was 92% versus 8% of individuals per subgroup. For the 150 min/week of MVPA cut-off, the distribution was 3.5% versus 96.5% of individuals per subgroup. However, information regarding these cut-offs can be found in the Supplementary Material (Supplementary Tables S1 and S2).

### 2.3. Subclinical Atherosclerosis Imaging

A Philips IU22 ultrasound system (Philips Healthcare, Bothell, WA, USA) was used to assess the presence of subclinical atherosclerosis in femoral and carotid vascular territories. Ultrasound images were acquired with linear high-frequency 2-dimensional probes (Philips Transducer L9-3, Philips Healthcare) using the Bioimage Study protocol for carotids [21] and a specifically designed protocol for femoral arteries [21]. Inspection sweeps were obtained at the right and left sides for the carotid (common, internal, external, and bulb) and femoral territories. A plaque was defined as a focal structure that protrudes into the lumen of the artery at least 0.5 mm or ≥50% thicker than the surrounding intima-media thickness. All measurements were analyzed using electrocardiogram-gated frames corresponding to end-diastole (R-wave) [22]. The presence of subclinical atherosclerosis was defined as the presence of at least one plaque in the carotid, femoral, or any territory (femoral and/or carotid territory). This methodology has been used and described previously [23].

### 2.4. Covariables and Definitions

Clinical and laboratory data were obtained during an annual medical examination. Biochemical measurements were performed on blood samples collected in fasting (>8 h) conditions. Total cholesterol, high-density lipoprotein cholesterol (HDL-c), triglycerides, and serum glucose concentrations were determined by enzyme analysis using the ILAB 650 analyzer from Instrumentation Laboratory (Bedford, MA, USA). Non-HDL-c was calculated by subtracting the HDL-c value from the total cholesterol value. Low-density lipoprotein cholesterol (LDL-c) was calculated using the Friedewald formula [24] when triglyceride levels were <400 mg/dL. Hypertension was defined as having systolic blood pressure ≥ 140 mmHg, diastolic blood pressure ≥ 90 mmHg, or self-reported use of blood pressure-lowering drugs [25]. Diabetes was defined as fasting plasma glucose ≥ 126 mg/dL, or self-reported use of glucose-lowering agents [25]. Dyslipidemia was defined as having a total cholesterol ≥ 240 mg/dL, LDL-c ≥ 160 mg/dL, HDL-c < 40 mg/dL or self-reported lipid-lowering drug use [26]. Obesity was defined as body mass index ≥ 30 kg/m^2^. Smoking habits were categorized as ever smoker (current and former smoker) if the participant reported having smoked in the last year, or having smoked at least 50 cigarettes in his lifetime, and never smoker. Sociodemographic characteristics included age.

### 2.5. Statistical Analysis

Data are presented as mean and standard deviation (SD) for continuous variables and percentages for categorical variables. Descriptive analyses were carried out for the overall sample and divided by PA groups. Presence of atherosclerosis in femoral arteries, in carotid arteries, or in any of both territories was fitted with logistic regression models depending on PA level at different intensities and adjusted for age, hypertension, dyslipidemia, diabetes, obesity and smoking status. Coefficients were used to calculate odds ratios (OR) for plaque presence of each PA level, using as reference the least active group in each case. *p*-values below 0.05 were considered statistically significant. Statistical analysis was performed using SPSS statistical software ver. 29.0 (IBM Corp, Armonk, NY, USA).

## 3. Results

The sample size included 428 participants with a mean age of 52.8 (SD 4.3) years. Compared with individuals who did not perform any VPA, those who performed more than 60 min/week were younger, had higher concentrations of HDL-c, and performed more MVPA. The prevalence of the presence of atheroma plaques among participants was 54.2% in the femoral territory and 38.1% in the carotid territory. At least one atheroma plaque in femoral or carotid territories was present in 69.2% of the overall sample (Table 1).

**Figure 1:**
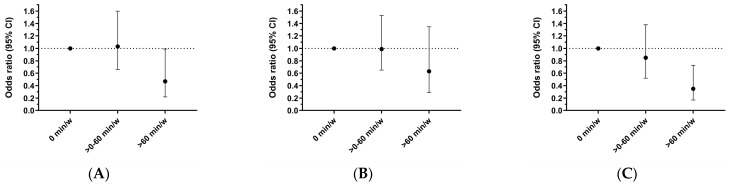
Odds ratio (95%CI) for atheroma plaque presence according to vigorous physical activity level group. (**A**) Odds ratio for femoral plaque. (**B**) Odds ratio for carotid plaque. (**C**) Odds ratio for any territory plaque (at least one plaque in the femoral or carotid territory).

The prevalence of the presence of atheroma plaques in the femoral territory in those who did not perform any VPA (0 min/week), those who performed between 0 and 60 min/week of VPA, and those who performed >60 min/week of VPA were 60.1%, 53.0%, and 33.3%, respectively (Table 1). The fully adjusted odds for having a femoral plaque were 0.47 (95%CI 0.22, 0.99, *p* < 0.05) for those who performed >60 min/week of VPA and 1.03 (95%CI 0.66, 1.60, *p* > 0.05) for those who performed between >0–60 min/week of VPA compared to those who did not perform VPA (0 min/week) (Table 2; Figure 1A).

The prevalence of the presence of atheroma plaques in the carotid territory in those who did not perform any VPA (0 min/week), those who performed between 0 and 60 min/week of VPA, and those who performed >60 min/week of VPA were, 41.5%, 37.4%, and 26.2%, respectively (Table 1). Although the odds for having a carotid plaque tended to be lower in those who performed >60 min/week of VPA in relation to those who did not perform VPA (0 min/week), (adjusted odds ratio 0.63; 95%CI: 0.29, 1.35, *p* > 0.05) statistical significance was not reached (Table 2; Figure 1B).

The prevalence of the presence of atheroma plaques in any analyzed territory (femoral and/or carotid) in those who did not perform any VPA (0 min/week), those who performed between 0 and 60 min/week of VPA, and those who perform >60 min/week of VPA were, 76.6%, 67.2%, and 45.2%, respectively (Table 1). The fully adjusted odds for having any atheroma plaque were 0.35 (95%CI 0.17, 0.73, *p* < 0.05) for those who performed >60 min/week of VPA and 0.85 (95%CI 0.52, 1.38, *p* > 0.05) for those who performed between >0–60 min/week of VPA compared to those who did not perform VPA (0 min/week) (Table 2; Figure 1C).

The odds of having a femoral, carotid, or any plaque (femoral and/or carotid) in relation to fulfilling the aerobic PA guidelines for adults based on the VPA threshold (75 min/week of VPA) [6] can be consulted in Supplementary Table S1.

**Figure 2:**
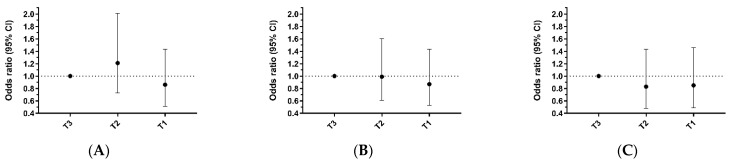
Odds ratio (95%CI) for atheroma plaque presence according to moderate to vigorous physical activity level group. (**A**) Odds ratio for femoral plaque. (**B**) Odds ratio for carotid plaque. (**C**) Odds ratio for any territory plaque (at least one plaque in femoral or carotid territory). T3: ≤340 min/week; T2: 340.01–497 min/week; T1: ≥497.01 min/week.

The prevalence of the presence of atheroma plaques in T3, T2, and T1 MVPA groups were 54.5%, 58.0%, and 50.0%, respectively, for femoral plaque; 39.9%, 39.2%, and 35.2%, respectively, for carotid plaque; and 72.0%, 68.5%, and 66.9%, respectively, for any territory (femoral and/or carotid) (Table 3). Although the fully adjusted odds for having an atheroma plaque in the femoral, carotid, or any territory tended to be lower in those who performed more MVPA (T1 group) in relation to those who performed less MVPA (T3 group), the differences were not statistically significant in any case (Table 3; Figure 2).

The odds of having a femoral, carotid, or any plaque (femoral and/or carotid) in relation to fulfilling the aerobic PA guidelines for adults based on the MVPA threshold (150 min/week of MVPA) [6] can be consulted in Supplementary Table S2.

## 4. Discussion

In this cross-sectional analysis conducted in middle-aged asymptomatic male workers, we demonstrate that only those who perform more than 60 min/week of VPA presented reduced odds for subclinical atherosclerosis in femoral or any analyzed vascular territories. It seems that high levels of MVPA could be insufficient to obtain the protective effect if no more than 1 h/week of VPA is performed. The most recent PA guidelines support that any PA produces health benefits, even LPA [6], but based on the results of this study, it seems that only VPA is effective to prevent atherosclerosis in adult men.

### 4.1. Physical Activity and Atherosclerosis

Previous studies that analyzed the role of PA in atherosclerosis disease found controversial results. Some of them showed the anti-atherosclerotic role of PA [7,8,9,10,11], but other studies conducted in athletes showed that higher doses of PA (a combination of high PA frequency, duration, and intensity) could increase the risk of presenting atheroma plaques [12,13]. However, it should be noted that athletes often present a very high PA level during the course of their lives in relation to the general population. This implies that athletes are exposed to high physiological stress due to high training loads, which could be detrimental to some parameters related to cardiovascular health, leading to atherosclerosis development [13]. However, the composition of atherosclerosis plaques presenting in athletes implies a lower CVD risk in relation to the atherosclerosis plaques that usually present in the general population [13].

Along the same line, a previous study that analyzed the relationship between PA and subclinical atherosclerosis in non-athletes showed how those individuals who completed three times the PA recommendation guidelines presented higher odds for atherosclerosis than those individuals who did not complete the PA recommendation guidelines [27]. However, the methodology used to assess PA through a total PA score presents some limitations, and also this study did not discriminate between PA intensities to reach the total PA level. Because of this, is it possible that the risk associated with a high level of PA could be due to the combination of high PA frequency and duration, but with a high proportion of this PA performed at very vigorous intensity, since based on our results, performing VPA for more than 1 h/week decreases the probability to present atherosclerosis.

In line with this assumption, a recent study conducted in middle-aged and older men that discriminates among different PA intensities reported that very vigorous PA (≥9 METs) increased the probability of presenting atherosclerosis [14]. However, on the other hand, this study also showed how performing PA at a vigorous intensity (6–9 METs) decreases the probability of presenting atherosclerosis plaques [14], results that are in line with those obtained in our study since only a few participants (n = 17) performed any PA at a very vigorous intensity (mean = 0.69 ± 5.98 min/week of very vigorous PA). Based on the overall scientific evidence, it could be possible that the relationship between PA intensity and atherosclerosis presents a U/J-shape association.

### 4.2. Methodology Used to Assess Physical Activity

As mentioned above, it should be noted that the methodology used to assess PA by most of the previous studies focused on analyzing the relationship between PA and atherosclerosis presents some limitations. Several studies used questionnaires to assess PA [12,14,27]. Although most of these questionnaires were previously validated, self-reported methods quantify PA from a subjective view of participants, and therefore, the quantification could be biased [28,29]. The methodology used to assess PA could condition the results obtained. A previous study showed that PA assessed by accelerometry was inversely associated with atherosclerosis, but if PA was assessed with subjective questionnaires, no association between PA and atherosclerosis was found [30]. In the present study, accelerometry was used to assess PA, which is an objective method that also presents some limitations, but the advantages, especially in establishing a dose–response relationship and quantifying intensity in an objective way, outweigh the limitations [29,31]. It could be possible that the controversial results regarding the association between PA and atherosclerosis may be, in part, a result of the methods used to assess PA. The present study significantly mitigates the constraints encountered in prior studies assessing PA. However, despite the high-quality methodology used to assess the PA level in this study, as well as in other previous studies that used different and more subjective methods to assess PA, the assessment was limited to the baseline, and PA can vary greatly during the life course.

### 4.3. Clinical Relevance

PA guidelines are developed based on high-quality scientific evidence, but from a wide health perspective [6]. Based on the results provided in this study, it could be possible that the minimum level of aerobic PA recommended for adult men (150 min/week of MVPA) could be insufficient to obtain a protective effect against atherosclerosis if no more than 60 min/week of VPA were performed. Although the results related to femoral plaques are significant but approach non-significance, the overall findings, which include plaque presence in both femoral and carotid territories, are robust and support this hypothesis.

On the other hand, as some studies have found that very vigorous PA increases atherosclerotic risk, it is difficult to state that increased PA intensity is always associated with health benefits from the perspective of atherosclerosis risk. Despite these limitations and knowing that atherosclerotic plaques increase the risk and are associated with cardiovascular events in the general population, performing more than 60 min/week of VPA (6–9 METs) seems to be key to decreasing the probability of presenting atherosclerosis plaques and to prevent future cardiovascular events.

### 4.4. Strengths and Limitations

The strengths of this study include the use of high-quality data collection methods to assess PA and subclinical atherosclerosis, and also the assessment of femoral vascular territory since other studies only analyze the carotid territory and the prevalence of subclinical atherosclerosis in the femoral territory is higher among middle-aged men [2]. However, several limitations should be acknowledged in our study. First, the cross-sectional analysis does not allow us to establish a causal, temporal link between the analyzed variables. Second, we only include Caucasian men in the study, so the results cannot be translated to other ethnicity or women and future research in other ethnicities and female population is needed to allow specific atherosclerosis probabilities. Third, the composition of atherosclerosis plaques was not analyzed and probably differences in plaque composition among the different PA groups analyzed could be showed. Fourth, despite the high-quality methodology used to assess the PA level, it was limited to a single baseline assessment, and PA can vary greatly during the life course. Future studies should aim to assess PA using accelerometry over extended periods to establish a dose–response relationship between PA and atherosclerosis.

## 5. Conclusions

In conclusion, it seems that performing more than 60 min/week of VPA is associated with reduced odds for subclinical atherosclerosis in femoral or any analyzed vascular territories in adult men. Although the minimum aerobic PA level recommended by worldwide PA guidelines for adults (150 min/week of MVPA) provides general health benefits, performing 60 min/week of VPA may offer a more protective effect against atherosclerosis in adult men.

## Figures and Tables

**Table 1:** Baseline characteristics of study participants according to level of physical activity.

		Vigorous Physical Activity Level (m/w)			
	Total	0	>0–60	>60	*p*-Value
N	428	188	198	42	
Age, years	52.8 (4.3)	53.9 (3.4)	52.1 (4.6)	51.5 (5.2)	<0.001 ^ab^
BMI, kg/m^2^	24.2 (3.3)	24.3 (3.4)	24.2 (3.3)	24.0 (2.9)	0.766
Systolic BP, mmHg	125.0 (13.9)	126.1 (14.7)	124.6 (13.7)	122.4 (10.7)	0.249
Diastolic BP, mmHg	82.0 (9.7)	83.0 (9.9)	81.6 (9.6)	79.9 (8.4)	0.132
Total cholesterol, mg/dL	217.2 (35.6)	218. 3 (34.7)	215.0 (36.6)	222.6 (34.3)	0.385
HDL-c, mg/dL	53.3 (11.4)	51.7 (10.1)	54.3 (12.3)	56.0 (11.2)	0.021
LDL-c, mg/dL	135.0 (29.7)	136.5 (30.5)	132.4 (28.8)	140.3 (29.6)	0.188
Triglycerides, mg/dL	148.6 (100.4)	152.4 (82.4)	148.6 (119.9)	131.5 (67.8)	0.475
Glucose, mg/dL	96.3 (18.7)	97.7 (18.9)	96.0 (19.4)	91.1 (13.4)	0.115
Hypertension, %	155 [36.2]	71 [37.8]	71 [35.9]	13 [31.0]	0.701
MVPA, min/week	443.2 (201.0)	371.1 (164.1)	470.2 (194.1)	639.3 (224.1)	<0.001 ^abc^
Dyslipidemia, %	190 [44.4]	95 [50.5]	79 [39.9]	16 [38.1]	0.076
Diabetes, %	26 [6.1]	11 [5.9]	13 [6.6]	2 [4.8]	0.893
Ever smokers, %	341 [79.7]	158 [84.0]	153 [77.3]	30 [71.4]	0.096
Obesity, %	22 [5.1]	13 [6.9]	8 [4.0]	1 [2.4]	0.307
Femoral plaque, %	232 [54.2]	113 [60.1]	105 [53.0]	14 [33.3]	0.006
Carotid plaque, %	163 [38.1]	78 [41.5]	74 [37.4]	11 [26.2]	0.175
Any plaque, %	296 [69.2]	144 [76.6]	133 [67.2]	19 [45.2]	<0.001

Notes: m/w: minutes/week; BMI: body mass index; BP: blood pressure; HDL-c: High-density lipoprotein cholesterol; LDL-c: Low-density lipoprotein cholesterol; MVPA: moderate to vigorous physical activity; any plaque: presence of at least one plaque in the femoral or carotid territory. Values are mean (SD) or number [%]. ^a^ Significant differences between 0 m/w and 0–60 m/w groups. ^b^ Significant differences between 0 m/w and >60 m/w groups. ^c^ Significant differences between 0–60 m/w and >60 m/w groups.

**Table 2:** Odds ratio (95%CI) for the presence of plaque in analyzed territories by vigorous physical activity level.

	**Vigorous Physical Activity Level**				
	**0 min/Week**	**>0–60 min/Week**	***p*-Value**	**>60 min/Week**	***p*-Value**
Number with femoral plaque/Total	113/188	105/198		14/42	
Unadjusted	1.00 (ref)	0.75 (0.50, 1.12)	0.161	0.33 (0.16, 0.67)	0.002
Age-adjusted	1.00 (ref)	0.95 (0.62, 1.46)	0.822	0.42 (0.20, 0.87)	0.020
Model 1	1.00 (ref)	1.03 (0.66, 1.60)	0.907	0.47 (0.22, 0.99)	0.048
Number with carotid plaque/Total	78/188	74/198		11/42	
Unadjusted	1.00 (ref)	0.84 (0.56, 1.27)	0.408	0.50 (0.24, 1.06)	0.069
Age-adjusted	1.00 (ref)	0.94 (0.62, 1.43)	0.759	0.57 (0.27, 1.21)	0.144
Model 1	1.00 (ref)	0.99 (0.65, 1.53)	0.989	0.63 (0.29, 1.35)	0.231
Number with any plaque/Total	144/188	133/198		19/42	
Unadjusted	1.00 (ref)	0.63 (0.40, 0.98)	0.041	0.25 (0.13, 0.51)	<0.001
Age-adjusted	1.00 (ref)	0.77 (0.48, 1.22)	0.263	0.31 (0.15, 0.64)	0.001
Model 1	1.00 (ref)	0.85 (0.52, 1.38)	0.512	0.35 (0.17, 0.73)	0.005

Notes: Model 1 Adjusted for age, hypertension, dyslipidemia, diabetes, obesity, and smoking status. Any plaque: presence of at least one plaque in femoral or carotid territory; ref: reference.

**Table 3:** Odds ratio (95%CI) for the presence of plaque in analyzed territories by moderate to vigorous physical activity level.

	**Moderate to Vigorous Physical Activity Level**				
	**T3**	**T2**	***p*-Value**	**T1**	***p*-Value**
Number with femoral plaque/Total	78/143	83/143		71/142	
Unadjusted	1.00 (ref)	1.15 (0.72, 1.84)	0.551	0.83 (0.52, 1.33)	0.443
Age-adjusted	1.00 (ref)	1.09 (0.67, 1.77)	0.732	0.79 (0.49, 1.29)	0.345
Model 1	1.00 (ref)	1.21 (0.73, 2.01)	0.453	0.86 (0.51, 1.43)	0.551
Number with carotid plaque/Total	57/143	56/143		50/142	
Unadjusted	1.00 (ref)	0.97 (0.61, 1.56)	0.904	0.82 (0.51, 1.33)	0.418
Age-adjusted	1.00 (ref)	0.94 (0.59, 1.52)	0.811	0.81 (0.50, 1.31)	0.380
Model 1	1.00 (ref)	0.99 (0.61, 1.60)	0.962	0.87 (0.53, 1.43)	0.590
Number with any plaque/Total	103/143	98/143		95/142	
Unadjusted	1.00 (ref)	0.85 (0.51, 1.41)	0.518	0.79 (0.47, 1.30)	0.348
Age-adjusted	1.00 (ref)	0.79 (0.47, 1.33)	0.372	0.76 (0.45, 1.28)	0.305
Model 1	1.00 (ref)	0.83 (0.48, 1.43)	0.501	0.85 (0.49, 1.46)	0.554

Notes: T3: ≤340 min/week of moderate to vigorous physical activity; T2: 340.01–497 min/week of moderate to vigorous physical activity; T1: ≥497.01 min/week of moderate to vigorous physical activity; any plaque: presence of at least one plaque in femoral or carotid territory; ref: reference. Model 1 Adjusted for age, hypertension, dyslipidemia, diabetes, obesity, and smoking status.

## Data Availability

The data presented in this study are available on request from the corresponding author. The data are not public due to ethical reasons.

## Supplementary Materials

The following supporting information can be downloaded at: https://www.mdpi.com/article/10.3390/jcm13175062/s1, Supplementary Table S1: Odds ratio (95%CI) for the presence of plaque in vascular territories by vigorous physical activity; Supplementary Table S2: Odds ratio (95%CI) for the presence of plaque in vascular territories by moderate to vigorous physical activity.
