# Supplementary material for: Atherosclerosis Prevalence among Different Physical Activity Patterns in Adult Men
Source: J Clin Med. 2024 Aug 26;13(17):5062. doi: 10.3390/jcm13175062 (PMC11395882; doi:10.3390/jcm13175062)
Supplement: Supplementary file 1 [file jcm-13-05062-s001.zip › jcm-3117307-supplementary.pdf]

Supplementary Table S1. Odds ratio (95%CI) for the presence of plaque in vascular territories by vigorous physical activity.

	Vigorous Physical Activity		
	**<75 min/week**	**≥75 min/week**	**p-value**
**Number with femoral plaque/Total**	222/395	10/33	
Unadjusted	1.00 (ref)	0.34 (0.16, 0.73)	0.006
Age-adjusted	1.00 (ref)	0.38 (0.17, 0.86)	0.020
Multivariable-adjusted 1	1.00 (ref)	0.39 (0.17, 0.89)	0.025
**Number with carotid plaque/Total**	153/395	10/33	
Unadjusted	1.00 (ref)	0.69 (0.32, 1.49)	0.340
Age-adjusted	1.00 (ref)	0.76 (0.35, 1.66)	0.489
Multivariable-adjusted 1	1.00 (ref)	0.80 (0.36, 1.75)	0.570
**Number with any plaque/Total**	281/395	15/33	
Unadjusted	1.00 (ref)	0.34 (0.17, 0.69)	0.003
Age-adjusted	1.00 (ref)	0.39 (0.18, 0.82)	0.013
Multivariable-adjusted 1	1.00 (ref)	0.39 (0.18, 0.83)	0.015

Model 1 Adjusted for age, hypertension, dyslipidemia, diabetes, obesity and smoking status.
Any plaque: presence of at least one plaque in femoral or carotid territory.

Supplementary Table S2. Odds ratio (95%CI) for the presence of plaque in vascular territories by moderate to vigorous physical activity.

	Moderate to Vigorous Physical Activity		
	<150 min/week	≥150 min/week	p-value
**Number with femoral plaque/Total**	8/15	224/413	
Unadjusted	1.00 (ref)	1.04 (0.37, 2.91)	0.945
Age-adjusted	1.00 (ref)	1.20 (0.41, 3.50)	0.740
Multivariable-adjusted 1	1.00 (ref)	1.38 (0.45, 4.28)	0.574
**Number with carotid plaque/Total**	7/15	156/413	
Unadjusted	1.00 (ref)	0.69 (0.25, 1.95)	0.488
Age-adjusted	1.00 (ref)	0.74 (0.26, 2.09)	0.564
Multivariable-adjusted 1	1.00 (ref)	0.82 (0.28, 2.37)	0.713
**Number with any plaque/Total**	11/15	285/413	
Unadjusted	1.00 (ref)	0.81 (0.25, 2.59)	0.722
Age-adjusted	1.00 (ref)	0.91 (0.28, 2.99)	0.875
Multivariable-adjusted 1	1.00 (ref)	1.16 (0.33, 4.09)	0.823

Model 1 Adjusted for age, hypertension, dyslipidemia, diabetes, obesity and smoking status.
Any plaque: presence of at least one plaque in femoral or carotid territory.
